# The sociodemographic patterning of sick leave and determinants of longer sick leave after mild and severe COVID-19: a nationwide register-based study in Sweden

**DOI:** 10.1093/eurpub/ckad191

**Published:** 2023-10-27

**Authors:** Malin Spetz, Yvonne Natt och Dag, Huiqi Li, Chioma Nwaru, Ailiana Santosa, Fredrik Nyberg, Maria Rosvall

**Affiliations:** Department of School of Public health and Community Medicine, Institute of Medicine, Sahlgrenska Academy at University of Gothenburg, Gothenburg, Sweden; Department of Clinical Microbiology, Sahlgrenska University Hospital, Region Västra Götaland, Gothenburg, Sweden; Department of School of Public health and Community Medicine, Institute of Medicine, Sahlgrenska Academy at University of Gothenburg, Gothenburg, Sweden; Department of School of Public health and Community Medicine, Institute of Medicine, Sahlgrenska Academy at University of Gothenburg, Gothenburg, Sweden; Department of School of Public health and Community Medicine, Institute of Medicine, Sahlgrenska Academy at University of Gothenburg, Gothenburg, Sweden; Department of School of Public health and Community Medicine, Institute of Medicine, Sahlgrenska Academy at University of Gothenburg, Gothenburg, Sweden; Department of School of Public health and Community Medicine, Institute of Medicine, Sahlgrenska Academy at University of Gothenburg, Gothenburg, Sweden; Department of School of Public health and Community Medicine, Institute of Medicine, Sahlgrenska Academy at University of Gothenburg, Gothenburg, Sweden; Department of Social Medicine, Regionhälsan, Region Västra Götaland, Sahlgrenska University Hospital, Gothenburg, Sweden

## Abstract

**Background:**

Studies on sociodemographic differences in sick leave after coronavirus disease 2019 (COVID-19) are limited and research on COVID-19 long-term health consequences has mainly addressed hospitalized individuals. The aim of this study was to investigate the social patterning of sick leave and determinants of longer sick leave after COVID-19 among mild and severe cases.

**Methods:**

The study population, from the Swedish multi-register observational study SCIFI-PEARL, included individuals aged 18–64 years in the Swedish population, gainfully employed, with a first positive polymerase chain reaction (PCR) test for severe acute respiratory syndrome coronavirus 2 (SARS-CoV-2) from 1 January 2020 until 31 August 2021 (*n* = 661 780). Using logistic regression models, analyses were adjusted for sociodemographic factors, vaccination, prior sick leave, comorbidities and stratified by hospitalization.

**Results:**

In total, 37 420 (5.7%) individuals were on sick leave due to COVID-19 in connection with their first positive COVID-19 test. Individuals on sick leave were more often women, older, had lower income and/or were born outside Sweden. These differences were similar across COVID-19 pandemic phases. The highest proportion of sick leave was seen in the oldest age group (10.3%) with an odds ratio of 4.32 (95% confidence interval 4.18–4.47) compared with the youngest individuals. Among individuals hospitalized due to COVID-19, the sociodemographic pattern was less pronounced, and in some models, even reversed. The intersectional analysis revealed considerable variability in sick leave between sociodemographic groups (range: 1.5–17.0%).

**Conclusion:**

In the entire Swedish population of gainfully employed individuals, our findings demonstrated evident sociodemographic differences in sick leave due to COVID-19. In the hospitalized group, the social patterning was different and less pronounced.

## Introduction

The impact of the coronavirus disease 2019 (COVID-19) pandemic, caused by severe acute respiratory syndrome coronavirus 2 (SARS-CoV-2), on public health is extensive. Although COVID-19 infection usually recedes within a few days or weeks, it can also take longer. Post-COVID patients have prolonged symptoms (≥12 weeks) and are often in need of extensive healthcare resources for treatment, rehabilitation and follow-up. Common persisting symptoms include fatigue, shortness of breath, muscle weakness, sleep disorder, anxiety and depression.^1–4^ Studies on COVID-19 long-term health consequences have mostly focused on the relatively small group of hospitalized patients, while more research is needed on long-term effects in the large group with milder disease.

In the working population, sick leave reflects health outcomes affecting workability and could serve as a proxy for the overall working population state of health. Previous research has demonstrated sociodemographic differences in sick leave in general,^5^ while studies focusing on COVID-19 are limited. In a recent Danish study of COVID-19 patients, admission to hospital, female sex, older age and comorbidity was associated with later return to work.^6^ These findings are in line with earlier research showing that the need for inpatient care and older age, but also sick leave prior to COVID-19, seem to be predictors of longer sick leave after COVID-19.^7^ Although long-term sick leave has been demonstrated to be relatively uncommon among COVID-19 patients followed in general practices, differences in length of sick leave with regard to sex, age and pre-existing conditions have been shown.^8^ Thus, more knowledge is needed to identify sociodemographic determinants associated with sick leave due to COVID-19 in a general population cohort, both after mild and severe disease.

The main objective of this study was to investigate sociodemographic differences in sick leave due to COVID-19, among gainfully employed individuals of working-age, in a fully population-based setting in both mild and severe cases. Our study expands on the results from the few published studies in the area by also taking into account factors related to vulnerability to COVID-19, e.g. social differences in work-related factors, vaccine coverage and prior comorbidities, and by using an intersectional analysis approach. An intersectional approach, considering several overlapping social factors at the same time to study heterogeneity between and within sociodemographic groups,^9–11^ has to our knowledge not previously been used in studies on sick leave. An additional objective was to study sociodemographic differences in sick leave of longer duration due to COVID-19.

## Methods

### Study design and study population

We used data from the SCIFI-PEARL (Swedish COVID-19 Investigation for Future Insights—a Population Epidemiology Approach using Register Linkage) observational study. As previously described,^12^ this is a nationwide research project addressing COVID-19 that encompasses multiple Swedish health and population registers linked on an individual level using the unique Swedish Personal Identity Number (PIN), which currently comprises the total Swedish population. The study cohort for the present analysis included all individuals aged 18–64 years, resident in Sweden on 1 January 2020 and gainfully employed, who had a first positive polymerase chain reaction (PCR) test for SARS-CoV-2 during the study period 1 January 2020 until 31 August 2021 (*n* = 661 780). In Sweden, sickness benefits are administered by the Swedish Social Insurance Agency (SSIA) and all gainfully employed individuals are entitled to sickness benefits. During the first 14 days of sick leave the employee receives sick pay from the employer, but for all sick leave episodes longer than 14 days, sickness benefit is paid by SSIA from day 15 and the episode from that day is available in the SSIA database. In the current study, the definition of sick leave was based on having received sickness benefits (see definition of outcome below), meaning that shorter sick leave periods (<15 days) were not included. Self-employed people, students, and the unemployed are also mostly eligible for sickness benefits, but with other rules for reimbursement and these groups are not covered by this study.

### Outcome

COVID-19-related sick leave was constructed using sick leave data from SSIA. The primary outcome was to have a sick leave period, here defined as starting an episode of receiving sickness benefits (i.e. ≥15 days sick leave), with COVID-19 as the registered reason, in connection with the individual’s first positive PCR test for SARS-CoV-2—defined as a time window encompassing 7 days before until 30 days after the test. The motivation for starting the time window up to 7 days before the positive PCR test was not to exclude cases likely connected to the positive test. The National Board of Health and Welfare has used a similar time window.^13^ Among those starting to receive sickness benefits due to COVID-19 within the time window, the length of the sick leave period was measured in days as secondary outcomes, i.e. to have a medium-to-long term (≥30 days) and long-term (≥12 weeks) sick leave due to a COVID-19 diagnosis. The 14 days of sick pay that preceded the SSIA-administered sickness benefits were added to the sick leave period to get the full duration. All individuals receiving sickness benefits in connection with the COVID-19 test were followed for at least 4 months to ensure enough follow-up from the first positive PCR test.

### Study exposures and covariates

Data on the sociodemographic exposure variables were acquired from the National Register of the Total Population (RTB) and the Longitudinal Integrated Database for Health Insurance and Labour Market Studies (LISA) from Statistics Sweden, including age, sex, income and country of birth. Age was categorized into three groups: 18–34, 35–49 and 50–64 years. Disposable income was divided into tertiles and then dichotomized into low income (1st tertile) and medium-high income (2nd and 3rd tertiles). In alignment with the World Bank classification, country of birth was categorized as Sweden, high-income countries and low- and middle-income countries (LMIC).^14^

In the intersectional analysis, a multi-categorical variable was constructed by making all possible combinations of the included sociodemographic factors—age (three categories), sex (two categories), income (two categories) and country of birth (three categories)—resulting in 36 intersectional strata.

Covariates included prior comorbidity diagnosed between 2015 and 2019, based on primary and secondary diagnoses registered with the International Classification of Diseases, version 10 (ICD-10) codes in the National Patient Register (NPR) from specialist outpatient visits and hospitalizations and include cardiovascular diseases (I00–I99), respiratory diseases (J00–J99), psychiatric diseases (F20–F39), cancer (C00–C97) and diabetes (E10, E11, E13, E14). Information on COVID-19 vaccination was obtained from the National Vaccination Register (NVR). Vaccination against COVID-19 was defined as receiving at least one dose before the individual’s first positive PCR test for COVID-19. Prior sick leave was defined as being on sick leave for ≥30 days for any diagnosis during the 3 months preceding the individual’s first positive PCR test for COVID-19. The definition of COVID-19 hospitalization in conjunction with the COVID-19 infection was being admitted to hospital at least 1 day with the COVID-19 ICD diagnosis U07 as the main diagnosis during the mentioned time window (encompassing 7 days before until 30 days after the individual’s first positive PCR test for Sars-CoV-2).

Information regarding occupation [Swedish Standard Occupational Classification (SSYK2012)] was obtained from LISA. The occupational codes were dichotomized into essential or non-essential occupations according to the definition by Nwaru *et al*.^15^ using criteria based on Billingsley *et al*.^16^ Essential occupations are those critical for societal function and where the work often needs to occur on-site at the workplace, e.g. working in healthcare, service sector or cleaning services.^15^

### Statistical analyses

In the descriptive analyses, proportions were used to describe differences in sociodemographic factors, comorbidities and other characteristics and evaluated with chi-square tests.

Crude and multivariable-adjusted odds ratios (ORs) with 95% confidence intervals (CI) were estimated for being on sick leave (yes/no) (primary outcome) and for having longer sick leave periods (secondary outcomes), i.e. ≥30 days (yes/no) and ≥12 weeks (yes/no), respectively, using logistic regression. Three different models were used. In the crude model 1, each sociodemographic variable was analyzed separately. In model 2, all sociodemographic variables were included and thus mutually (i.e. simultaneously) adjusted for. In model 3, further adjustments for the additional study covariates (i.e. comorbidities, prior sick leave, vaccination and hospitalization) were made. These models were used for analyses of the entire study population and also for stratified analyses based on hospitalization due to COVID-19. In the intersectional analysis, ORs for being on sick leave were estimated for each intersectional stratum relative to one reference stratum. Sensitivity analyses were performed excluding those who died or emigrated within 4 months from their first positive test for SARS-CoV-2 and stratifying for pandemic phases when the index COVID-19 infection occurred, respectively. The first pandemic phase with the spread of SARS-CoV-2 in Sweden was defined, by the National Board of Health and Welfare, to occur between 1 January 2020 and 18 October 2020, the second phase between 19 October 2020 and 31 January 2021 and the third phase between 1 February 2021 and 31 August 2021.^17^ Sensitivity analysis was also made adjusting for on-site work, i.e. essential or non-essential occupations. The statistical packages used included IBM SPSS Statistics for windows version 26.0 and Stata 17.0.

## Results

The study population included 661 780 individuals aged 18–64 years, of whom 5.7% were on sick leave due to COVID-19 in connection with a first positive COVID-19 PCR test (table 1). In comparison with individuals not on sick leave, individuals on sick leave were more often women, older, had a lower income, were born outside Sweden and/or had a history of cardiovascular disease, respiratory disease, cancer or diabetes. Furthermore, hospitalization due to COVID-19 was more common in the group on sick leave. Prior vaccination with at least one dose of a COVID-19 vaccine and prior sick leave were, however, somewhat more common among individuals not on sick leave.

**Table 1:** Distribution (%) of sociodemographic factors, comorbidities, vaccination, prior sick leave and hospitalization among individuals in the Swedish population with a first positive PCR test for SARS-CoV-2, aged 18–64 years and gainfully employed, by sick leave due to COVID-19

	Sick leave	No sick leave	*P*-value	Total
	*N* = 37 420 (5.7%)	*N* = 624 360 (94.3%)		*N* = 661 780
Sex				
Men	40.1%	48.1%	<0.001	47.7%
Women	59.9%	51.9%		52.3%
Age group (years)				
18–34	14.6%	37.2%	<0.001	35.9%
35–49	35.7%	37.0%		37.0%
50–64	49.6%	25.8%		27.2%
Income^a^				
Medium-high	65.3%	67.1%	<0.001	67.0%
Low	34.7%	32.9%		33.0%
Country of birth^b^				
Sweden	68.2%	79.6%	<0.001	78.9%
HIC	6.4%	4.7%		4.8%
LMIC	25.4%	15.8%		16.3%
Comorbidities (2015–19)				
Cardiovascular disease	3.9%	2.1%	<0.001	2.2%
Respiratory disease	3.5%	1.9%	<0.001	2.0%
Cancer	1.0%	0.8%	<0.001	0.8%
Psychiatric disease	1.1%	1.1%	0.250	1.1%
Diabetes	1.7%	0.9%	<0.001	0.9%
Vaccination^c^	2.0%	4.2%	<0.001	4.1%
Prior sick leave	1.6%	2.7%	<0.001	2.0%
Hospitalization	22.5%	1.4%	<0.001	2.6%

a Disposable income: medium/high: 2nd and 3rd tertiles, low: 1st tertile.

b Country of birth: Sweden, HIC: high-income countries; MIC: middle-income countries; LIC: low-income countries.

c Vaccinated with at least one dose of a COVID-19 vaccine before the first positive PCR test for COVID-19.

The number of sick leave episodes followed the daily number of positive tests for COVID-19 relatively well, with a total cumulative number of 37 420 sick leave episodes over the study period (Supplementary figure S1).

The OR for sick leave was higher among older age groups, women, low-income groups and those born outside Sweden (table 2). In the oldest age group, 10.3% were on sick leave due to COVID-19 and in the crude model 1, the OR of sick leave in this group was 4.88 (95% CI 4.73–5.03). In the adjusted models 2 and 3, the ORs remained essentially unchanged. Sensitivity analyses excluding those who died or emigrated within 4 months from their first positive test for SARS-CoV-2 showed similar results (data not shown). Sensitivity analysis stratifying by pandemic phases showed a somewhat higher presence of COVID-related sick leave for individuals in the first phase (10.9%) compared with the second phase (6.7%) and third phase (3.6%). However, the sociodemographic pattern was similar for all three phases (Supplementary table S1).

**Table 2:** ORs and 95% CI of sick leave due to COVID-19 in the total population and among those not hospitalized or hospitalized

Characteristics	Sick leave, %^a^	Model 1,^b^ OR (95% CI)	Model 2,^c^ OR (95% CI)	Model 3,^d^ OR (95% CI)
Total population, *n* = 661 780				
Sex				
Men	4.8	1.00	1.00	1.00
Women	6.5	1.39 (1.36–1.42)	1.38 (1.35–1.41)	1.72 (1.68–1.76)
Age group (years)				
18–34	2.3	1.00	1.00	1.00
35–49	5.5	2.45 (2.37–2.52)	2.37 (2.29–2.45)	2.20 (2.13–2.28)
50–64	10.3	4.88 (4.73–5.03)	5.25 (5.09–5.42)	4.32 (4.18–4.47)
Income^e^				
Medium-high	5.5	1.00	1.00	1.00
Low	6.0	1.09 (1.06–1.11)	1.28 (1.25–1.31)	1.29 (1.25–1.32)
Country of birth^f^				
Sweden	4.9	1.00	1.00	1.00
HIC	7.5	1.60 (1.53–1.67)	1.38 (1.32–1.45)	1.28 (1.23–1.35)
LMIC	8.8	1.88 (1.84–1.93)	1.87 (1.82–1.92)	1.56 (1.51–1.60)
Non-hospitalized, *n* = 644 894		Model 1,^b^ OR (95% CI)	Model 2,^c^ OR (95% CI)	Model 3,^g^ OR (95% CI)
Sex				
Men	3.2	1.00	1.00	1.00
Women	5.7	1.82 (1.77–1.86)	1.79 (1.74–1.83)	1.83 (1.79–1.88)
Age group (years)				
18–34	2.1	1.00	1.00	1.00
35–49	4.5	2.23 (2.16–2.31)	2.17 (2.10–2.25)	2.20 (2.13–2.28)
50–64	7.9	4.08 (3.95–4.22)	4.45 (4.30–4.61)	4.58 (4.42–4.74)
Income^e^				
Medium-high	4.3	1.00	1.00	1.00
Low	4.9	1.17 (1.14–1.19)	1.35 (1.32–1.39)	1.36 (1.33–1.40)
Country of birth^f^				
Sweden	3.9	1.00	1.00	1.00
HIC	5.8	1.51 (1.44–1.59)	1.31 (1.25–1.38)	1.31 (1.24–1.38)
LMIC	6.8	1.77 (1.72–1.82)	1.73 (1.67–1.78)	1.72 (1.67–1.77))
Hospitalized, *n* = 16 886		Model 1,^b^ OR (95% CI)	Model 2,^c^ OR (95% CI)	Model 3,^g^ OR (95% CI)
Sex				
Men	49.7	1.00	1.00	1.00
Women	50.0	1.01 (0.95–1.08)	1.05 (0.98–1.12)	1.14 (1.06–1.21)
Age group (years)				
18–34	33.5	1.00	1.00	1.00
35–49	47.8	1.82 (1.63–2.03)	1.79 (1.60–2.00)	1.85 (1.66–2.08)
50–64	54.3	2.36 (2.13–2.62)	2.19 (1.97–2.44)	2.41 (2.16–2.69)
Income^e^				
Medium-high	52.6	1.00	1.00	1.00
Low	44.1	0.71 (0.67–0.76)	0.80 (0.74–0.85)	0.85 (0.79–0.91)
Country of birth^f^				
Sweden	50.7	1.00	1.00	1.00
HIC	54.4	1.16 (1.02–1.31)	1.14 (1.00–1.29)	1.13 (0.99–1.29)
LMIC	46.7	0.85 (0.80–0.91)	0.94 (0.88–1.01)	0.88 (0.82–0.95)

a Sick leave due to COVID-19.

b Model 1: Crude ORs models for each sociodemographic factor.

c Model 2: Mutually adjusted.

d Model 3: Model 2 + adjusted for comorbidities, prior sick leave, vaccination and hospitalization.

e Disposable income: medium/high: 2nd and 3rd tertile, low: 1st tertile.

f Country of birth: Sweden, HIC: high-income countries; LMIC: low- and middle-income countries.

g Model 3: Model 2 + adjusted for comorbidities, prior sick leave and vaccination.

In stratified analyses based on hospitalization, the proportion on sick leave due to COVID-19 was considerably higher among hospitalized than among non-hospitalized individuals (table 2). The overall sociodemographic risks for COVID-related sick leave were essentially the same among the large group of non-hospitalized individuals as for the total population, as might be expected. Among hospitalized and non-hospitalized individuals, female sex and older age were associated with higher ORs of sick leave, but the associations were weaker in the smaller hospitalized group. In the hospitalized group, the pattern of association between country of birth and income and being on sick leave was more or less the inverse of the association among non-hospitalized (table 2). Sensitivity analysis taking into account on-site work by additionally adjusting model 3 for essential or non-essential occupations did not change the pattern of associations (data not shown).

**Figure 1:**
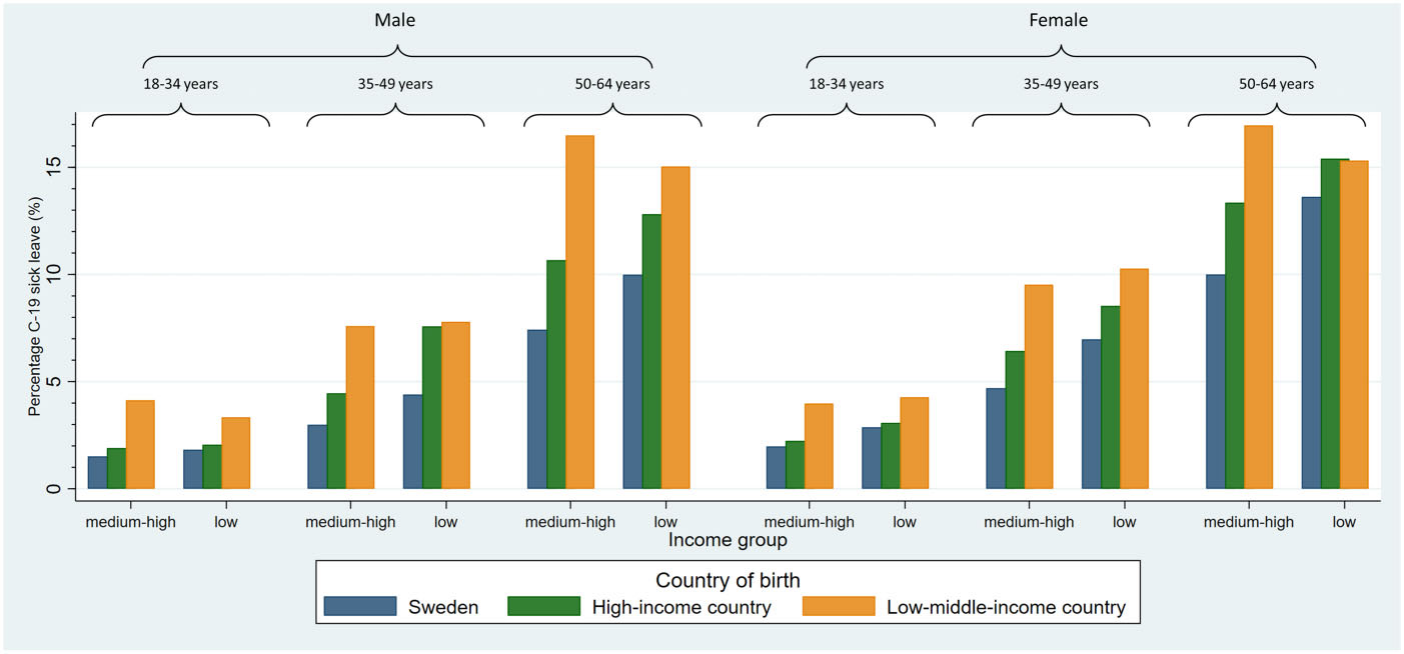
Proportions of individuals on sick leave due to COVID-19 in 36 intersectional strata defined by sex (men, women), age groups (18–34, 35–49 and 50–64 years), country of birth (Sweden; high-income countries; low-middle-income countries) and income [low income (1st tertile); medium/high income (2nd and 3rd tertile)]

The intersectional analysis, including 36 sociodemographic strata, demonstrated large variations between the different groups defined by cross-classification of sociodemographic characteristics in being on sick leave due to COVID-19 (figure 1, Supplementary table S2), from 1.5% among men aged 18–34 years, with a medium-high income and born in Sweden, to 17.0% among women aged 50–64 years, with a medium-high income and born in an LMIC.

The median duration of the sick leave period (including the 14 days of sick pay) was 31 days. Female sex, older age, having a low income or being born outside Sweden were all factors associated with higher ORs of medium-to-long-term sick leave (i.e. duration of ≥30 days) (table 3). Regarding long-term sick leave (≥12 weeks), the pattern was generally similar, except for the association with country of birth, where those born in a LMIC had lower odds of longer sick leave. Sensitivity analyses excluding those who died or emigrated within 4 months from their first positive test for SARS-CoV-2 demonstrated similar results (data not shown). Similar to sick leave in general, both sick leave for ≥30 days and ≥12 weeks were much more common among hospitalized individuals (table 3). The overall social pattern for longer sick leave seen in the total population was generally similar to that seen in the largest subgroup, non-hospitalized individuals. Among hospitalized individuals, female sex and higher age were clearly associated with higher ORs for both ≥30 days and ≥12 weeks of sick leave. However, low income and being born in an LMIC were associated with lower ORs for sick leave ≥30 days and being born in a LMIC was associated with lower OR of sick leave ≥12 weeks. Sensitivity analysis, additionally adjusting model 3 for essential or non-essential occupations, showed a very similar sociodemographic pattern (data not shown).

**Table 3:** ORs with 95% CI of sick leave ≥30 days and ≥12 weeks due to COVID-19 in the total population and among those non-hospitalized or hospitalized

Charachteristics	Sick leave 30 days, %^a^	Model 1,^b^ OR (95% CI)	Model 2,^c^ OR (95% CI)	Model 3,^d^ OR (95% CI)	Sick leave 12 weeks, %^a^	Model 1,^b^ (95% CI)	Model 2,^c^ (95% CI)	Model 3,^d^ (95% CI)
Total population, *n* = 661 780								
Sex								
Men	2.5	1.00	1.00	1.00	0.6	1.00	1.00	1.00
Women	3.4	1.37 (1.34–1.41)	1.37 (1.33–1.41)	1.82 (1.77–1.88)	0.8	1.32 (1.24–1.40)	1.31 (1.23–1.39)	1.85 (1.74–1.98)
Age group (years)								
18–34	1.0	1.00	1.00	1.00	0.2	1.00	1.00	1.00
35–49	2.8	2.86 (2.73–3.00)	2.80 (2.67–2.94)	2.50 (2.38–2.63)	0.6	3.40 (3.06–3.78)	3.44 (3.09–3.83)	2.80 (2.51–3.13)
50–64	5.8	6.18 (5.91–6.47)	6.69 (6.38–7.00)	4.88 (4.65–5.13)	1.4	7.52 (6.79–8.33)	8.06 (7.25–8.95)	4.43 (3.97–4.94)
Income^e^								
Medium-high	2.9	1.00	1.00	1.00	0.7	1.00	1.00	1.00
Low	3.1	1.06 (1.03–1.09)	1.32 (1.27–1.36)	1.31 (1.26–1.35)	0.6	0.95 (0.89–1.01)	1.29 (1.21–1.39)	1.23 (1.15–1.32)
Country of birth^f^								
Sweden	2.6	1.00	1.00	1.00	0.6	1.00	1.00	1.00
HIC	4.1	1.62 (1.52–1.71)	1.38 (1.30–1.46)	1.23 (1.15–1.31)	1.0	1.54 (1.37–1.73)	1.30 (1.15–1.47)	1.06 (0.93–1.20)
LMIC	4.5	1.78 (1.72–1.84)	1.74 (1.68–1.81)	1.30 (1.25–1.36)	0.8	1.24 (1.14–1.33)	1.20 (1.11–1.30)	0.73 (0.67–0.79)
Non-hospitalization,		Model 1,^b^	Model 2,^c^	Model 3^g^,		Model 1,^b^	Model 2,^c^	Model 3^g^,
*n* = 644 894		OR (95% CI)	OR (95% CI)	OR (95% CI)		OR (95% CI)	OR (95% CI)	OR (95% CI)
Sex								
Men	1.4	1.00	1.00	1.00	0.2	1.00	1.00	1.00
Women	2.7	2.04 (1.96–2.11)	2.00 (1.92–2.07)	2.03 (1.96–2.11)	0.5	2.78 (2.52–3.05)	2.72 (2.47–2.98)	2.76 (2.51–3.03)
Age group (years)								
18–34	0.8	1.00	1.00	1.00	0.1	1.00	1.00	1.00
35–49	2.1	2.54 (2.41–2.67)	2.51 (2.38–2.65)	2.53 (2.40–2.67)	0.4	2.85 (2.52–3.23)	2.94 (2.59–3.33)	2.95 (2.60–3.35)
50–64	3.8	4.74 (4.50–4.99)	5.24 (4.97–5.52)	5.34 (5.06–5.63)	0.6	4.44 (3.93–5.02)	4.84 (4.26–5.49)	4.82 (4.24–5.48)
Income^e^								
Medium-high	2.0	1.00	1.00	1.00	0.4	1.00	1.00	1.00
Low	2.3	1.18 (1.14–1.22)	1.43 (1.38–1.49)	1.44 (1.38–1.50)	0.4	1.10 (1.01–1.20)	1.42 (1.29–1.55)	1.41 (1.29–1.55)
Country of birth^f^								
Sweden	1.9	1.00	1.00	1.00	0.4	1.00	1.00	1.00
HIC	2.8	1.51 (1.40–1.62)	1.28 (1.19–1.38)	1.28 (1.19–1.37)	0.5	1.39 (1.18–1.63)	1.17 (0.99–1.38)	1.16 (0.99–1.37)
LMIC	3.0	1.60 (1.53–1.67)	1.53 (1.46–1.59)	1.52 (1.45–1.59)	0.3	0.88 (0.78–0.99)	0.82 (0.72–0.92)	0.81 (0.72–0.91)
Hospitalization,		Model 1,^b^	Model 2,^c^	Model 3,^g^		Model 1,^b^	Model 2,^c^	Model 3,^g^
*n* = 16 886		OR 95%CI	OR 95%CI	OR 95%CI		OR 95%CI	OR 95%CI	OR 95%CI
Sex								
Men	31.5	1.00	1.00	1.00	11.7	1.00	1.00	1.00
Women	38.4	1.15 (1.08–1.23)	1.19 (1.11–1.27)	1.27 (1.18–1.35)	12.9	1.12 (1.02–1.23)	1.15 (1.04–1.26)	1.18 (1.07–1.30)
Age group (years)								
18–34	20.5	1.00	1.00	1.00	5.3	1.00	1.00	1.00
35–49	32.7	1.89 (1.67–2.14)	1.91 (1.68–2.16)	1.95 (1.72–2.22)	10.0	1.97 (1.58–2.45)	2.07 (1.66–2.59)	2.11 (1.69–2.64)
50–64	41.7	2.77 (2.46–3.13)	2.67 (2.37–3.02)	2.86 (2.53–3.24)	14.7	3.05 (2.48–3.76)	3.04 (2.45–3.77)	3.16 (2.54–3.92)
Income^e^								
Medium-high	38.7	1.00	1.00	1.00	13.1	1.00	1.00	1.00
Low	31.8	0.74 (0.69–0.79)	0.86 (0.80–0.93)	0.91 (0.84–0.98)	10.2	0.76 (0.68–0.84)	0.97 (0.86–1.08)	1.00 (0.89–1.12)
Country of birth^f^								
Sweden	37.6	1.00	1.00	1.00	13.5	1.00	1.00	1.00
HIC	39.9	1.11 (0.97–1.23)	1.06 (0.93–1.21)	1.05 (0.92–1.20)	13.3	0.99 (0.82–1.19)	0.94 (0.78–1.14)	0.93 (0.78–1.13)
LMIC	33.1	0.82 (0.77–0.88)	0.90 (0.84–0.97)	0.86 (0.79–0.92)	9.2	0.65 (0.59–0.73)	0.70 (0.62–0.78)	0.67 (0.60–0.76)

a Sick leave due to COVID-19.

b Model 1: Crude ORs models for each sociodemographic factor.

c Model 2: Mutually adjusted.

d Model 3: Model 2 + adjusted for comorbidities, prior sick leave, vaccination and hospitalization.

e Disposable income: Medium/high: 2nd and 3rd tertile, Low: 1st tertile.

f Country of birth: Sweden, HIC: high-income countries; LMIC: low- and middle-income countries.

g Model 3: Model 2 + adjusted for comorbidities, prior sick leave and vaccination.

## Discussion

This nationwide register-based study demonstrated considerable sociodemographic differences in sick leave due to COVID-19. The sociodemographic pattern was similar across the different COVID-19 pandemic phases. Among non-hospitalized individuals, the overall social patterning of sick leave was similar for the total population, as might be expected since they constituted the vast majority of the COVID-19 cases. However, among individuals hospitalized due to COVID-19, sick leave was more common and the sociodemographic pattern was less pronounced.

Our findings align with previous studies showing longer sick leave periods after COVID-19 among women and older age groups. A Danish study demonstrated that older age and female sex were associated with a reduced chance to return to work after a COVID-19 infection.^6^ Additionally, in a previous Swedish register-based study with a follow-up until 31 August 2020, older age was a predictor of longer sick leave,^7^ and older age has also been associated with long-term sick leave among individuals of working-age followed in general practices.^8^ Older age is a well-known risk factor for severe COVID-19^18^^,^^19^ and these findings are, therefore in part expected. The interpretation of results indicating that women are more frequently on sick leave than men is more complex. Previous research has demonstrated that men have a higher risk of severe COVID-19.^19^ Nevertheless, our results confirm the findings by Jacobsen et al. showing that women had a lower chance of returning to work.^6^ The results also align with a previous Swedish study demonstrating that more women than men tended to have longer sick leave periods due to COVID-19.^7^ Swedish women overall more often have sick leave than men,^20^ and according to statistics from SSIA, sickness episodes due to COVID-19 are also more common among women,^21^ as was also shown in the present study. Thus, sex differences in sickness absence probably reflect more complex aspects of inability to work that are at least partly beyond factors specifically related to COVID-19.

Individuals with low income or born outside Sweden were more often on sick leave due to COVID-19. The differences in sick leave could theoretically, at least to some extent, be explained by factors related to characteristics and context associated with various occupations. People working in essential occupations have a higher risk of COVID-19 because of their greater exposure to the virus.^15^^,^^22^ Earlier research also indicates that among essential workers who could not work remotely, also called frontline workers, lower-income groups and certain ethnic minorities were overrepresented.^23^ However, in our study, sensitivity analysis demonstrated an overall similar pattern of sociodemographic differences in sick leave after adjustment for holding an essential occupation or not. The lower ORs for long-term sick leave (≥12 weeks) among those born in LMIC might theoretically be due to fear of losing their job, considering that this group is more likely to have temporary employment as compared with those born in Sweden and high-income workers.^24^

In our study, addressing a gainfully employed working-age population, relatively few individuals were hospitalized. COVID-19 patients admitted to hospitals more often had longer sick leave periods. These findings are in line with previous research showing a lower probability of returning to work within 3 months from a positive test among hospitalized individuals with COVID-19,^6^ and that the need for inpatient care seems to be a predictor of longer sick leave.^7^ Among hospitalized individuals, the sociodemographic differences in sick leave were generally less pronounced and regarding country of birth and income, the associations were even reversed. These results are in line with a relatively recent meta-analysis concluding that even if the risk of COVID-19 is higher in certain ethnic groups, there were overall no obvious differences in outcome (i.e. severe disease, admission to an ICU and death) between ethnic groups hospitalized due to COVID-19.^25^ Comorbidities such as cardiovascular or respiratory disease are well-known factors affecting the severity of COVID-19.^19^ In our study, a history of cardiovascular disease, respiratory disease, cancer or diabetes was also more common among individuals on sick leave, reflecting a vulnerability to COVID-19 in this group. However, the sociodemographic differences in sick leave persisted after adjusting for such comorbidities.

Another factor affecting the severity of COVID-19 disease is vaccination. Previous research, including our studies, has demonstrated sociodemographic differences in COVID-19 vaccination uptake in the working-age population, including a lower coverage among younger age groups, men, lower-income groups, and individuals born outside Sweden.^11^ In the present study, vaccination was more common among individuals not on sick leave. This may potentially reflect that vaccination protects against more severe COVID-19 disease, which might result in a lesser effect on work capacity. Further investigation of this topic is outside the scope of this study. Nevertheless, adjustment for vaccination did not alter the associations between sociodemographic factors and sick leave.

The results for individual risk factors presented are based on group averages; however, it is important to bear in mind that the sociodemographic groups used are not homogenous and that there might be heterogeneity in sick leave within groups. For example, in the older age group 50–64 years, the proportion on sick leave in different intersectional subgroups ranged from 7.4% to 17.0% (mean: 10.3%), while the proportion in women ranged from 2.0% to 17.0% (mean: 6.5%). Our findings indicate, consistent with previous research, that a wider variability may be revealed when considering multiple sociodemographic determinants at the same time in comparison to when focusing on one variable at a time and group average risks.^9–11^

The additional sensitivity analysis stratified by pandemic phases demonstrated a somewhat higher proportion of individuals on sick leave after COVID-19 in the first phase than in the second and third phases, but the overall sociodemographic pattern for sick leave was essentially the same for all three phases. The relatively higher level of sick leave for individuals with COVID-19 in the first phase may reflect that at the beginning of the pandemic, the PCR test capacity was limited, individuals with more severe disease were prioritized, the SARS-CoV-2-virus was more virulent, and no COVID-19 vaccines were yet available.

Our study has several important strengths. By including the total Swedish population aged 18–64 years, the findings are highly representative and generalizable. Secondly, the Swedish PIN enables multiple register linkages with very high accuracy (essentially error-free) and very few missing data. Thirdly, the Swedish registers used are nationwide and of recognized high quality, and the data on positive SARS-CoV-2 tests are extremely reliable since reporting of COVID-19 is regulated by Swedish law. One study limitation is that individuals with mild symptoms could be missed at the beginning of the pandemic due to a limited test capacity. However, separate analyses for the different pandemic phases did not alter our findings regarding the social patterning in sick leave. Another limitation is that sick leave shorter than 15 days was not included in the analysis, and thus sociodemographic differences in short-term sick leave are not covered by this study.

In conclusion, in an analysis covering the entire Swedish population of gainfully employed individuals aged 18–64 years with a first positive PCR test for SARS-CoV-2, sick leave due to COVID-19 was relatively more common among women, in older age groups, among those with lower income and born outside Sweden. In the hospitalized COVID-19 group, sick leave after COVID-19 was more common, and the sociodemographic pattern was less pronounced and, in some models, even reversed. Finally, the intersectional analysis revealed considerable sociodemographic heterogeneity in sick leave within broader one-factor sociodemographic groups. The findings are relevant from both a public health and a healthcare perspective.

## Supplementary Material

ckad191_Supplementary_Data

## Data Availability

The data in this study are pseudonymized individual level data from Swedish healthcare registers and are not publicly available according to Swedish legislation. They can be obtained from the respective Swedish public data holders on the basis of ethics approval for the research in question, subject to relevant legislation, processes and data protection.
