# Supplementary material for: The sociodemographic patterning of sick leave and determinants of longer sick leave after mild and severe COVID-19: a nationwide register-based study in Sweden
Source: Eur J Public Health. 2023 Oct 27;34(1):121–8. doi: 10.1093/eurpub/ckad191 (PMC10843940; doi:10.1093/eurpub/ckad191)
Supplement: ckad191_Supplementary_Data [file ckad191_supplementary_data.zip › ckad191_Supplementary_Data/ejph-2023-06-om-0328-File005.pdf]

Supplementary Table 1 Adjusted odds ratios (ORs) with 95% Confidence Intervals (CI) of sick leave due to Covid-19 in the total population during the first, second and third pandemic phase

Characteristics	Sick leave %[c]	Model 1[d], OR 95%CI	Model 2[e], OR 95%CI	Model 3[f], OR 95%CI
**Pandemic phase 1,** n= 63 231				
Sex				
Men	10.4	1.00	1.00	1.00
Women	11.3	1.10 (1.04-1.15)	1.09 (1.03-1.15)	1.57 (1.48-1.66)
Age group (years)				
18-34	4.9	1.00	1.0	1.00
35-49	11.2	2.45 (2.28-2.64)	2.29 (2.12-2.47)	2.01 (1.86-2.18)
50-64	18.1	4.31(4.01-4.62)	4.53 (4.21-4.88)	3.28 (3.04-3.55)
Income[a]				
Medium-High	10.6	1.00	1.00	1.00
Low	11.7	1.12 (1.09-1.19)	1.27 (1.19-1.34)	1.24 (1.16-1.32)
Country of birth[b]				
Sweden	9.0	1.00	1.00	1.00
HIC	15.1	1.81 (1.63-2.01)	1.51 (1.36-1.68)	1.33 (1.19-1.50)
LMIC	17.1	2.10 (1.98-2.22)	1.99 (1.88-2.12)	1.55 (1.45-1.65)
**Pandemic phase 2,** n= 285 553		Model 1[d], OR 95%CI	Model 2[e], OR 95%CI	Model 3[f], OR 95%CI
Sex				
Men	5.4	1.00	1.00	1.00
Women	7.9	1.49 (1.44-1.53)	1.47 (1.42-1.51)	1.64 (1.59-1.70)
Age group (years)				
18-34	3.0	1.00	1.00	1.00
35-49	6.6	2.31 (2.21-2.41)	2.27 (2.17-2.37)	2.18 (2.09-2.28)
50-64	11.6	4.29 (4.11-4.47)	4.69 (4.48-4.90)	4.19 (4.00-4.38)
Income[a]				
Medium-High	6.5	1.00	1.00	1.00
Low	7.2	1.13 (1.09-1.16)	1.35 (1.30-1.40)	1.35 (1.30-1.40)
Country of birth[b]				
Sweden	5.9	1.00	1.00	1.00
HIC	8.5	1.49 (1.40-1.58)	1.30 (1.22-1.38)	1.24 (1.16-1.32)
LMIC	9.6	1.70 (1.64-1.76)	1.67 (1.61-1.73)	1.52 (1.47-1.58)
**Pandemic phase 3,** n=312 996		Model 1[d], OR 95%CI	Model 2[e], OR 95%CI	Model 3[f], OR 95%CI
Sex				
Men	3.3	1.00	1.00	1.00
Women	4.0	1.23 (1.18-1.27)	1.24 (1.19-1.29)	1.67 (1.60-1.75)
Age group (years)				
18-34	1.2	1.00	1.00	1.00
35-49	3.5	2.99 (2.81-3.19)	2.93 (2.75-3.12)	2.58 (2.42-2.76)
50-64	7.2	6.50 (6.12-6.90)	6.95 (6.53-7.39)	5.20 (4.87-5.55)
Income[a]				
Medium-High	3.6	1.00	1.00	1.00
Low	3.7	1.02 (0.98-1.07)	1.26 (1.20-1.31)	1.27 (1.21-1.33)
Country of birth[b]				
Sweden	3.2	1.00	1.00	1.00
HIC	5.0	1.61 (1.49-1.74)	1.39 (1.28-1.50)	1.31 (1.20 -1.43)
LMIC	5.6	1.80 (1.72-1.89)	1.79 (1.71-1.88)	1.39 (1.32-1.47)

a. Disposable income: Medium/High: 2nd and 3rd tertile, Low: 1st tertile.
b. Country of birth: Sweden, HIC: High Income Countries; LMIC: Low- and Middle Income Countries.
c. Sick leave due to Covid-19.

d. Model 1: Crude odds ratios models for each sociodemographic factor.
e. Model 2: Mutually adjusted.
f. Model 3: Model 2 + adjusted for comorbidities, prior sick leave, vaccination and hospitalisation.

Supplementary Table 2 Adjusted odds ratios (ORs) with 95% Confidence Interval (CI) of sick leave by intersectional strata based on sociodemographic factors i.e., age, sex, income and country of birth.

Intersectional strata	N	Sick leave[c]	Model 4
		%	OR (95% CI)[d]
Men/Med-High inc[a]/ Born Swe[b]/Age 18-34	62 269	1,5%	1·00[e]
Men/Low inc/Born Swe/Age 18-34	28414	1,8%	1,21 (1,08-1,34)
Men/Med High inc/Born HIC/Age 18-34	1845	1,9%	1,26 (0,89-1,77)
Women/Med-High inc/Born Swe/Age 18-34	55887	2,0%	1,31 (1,20-1,43)
Men/Low inc/Born HIC/Age 18-34	1557	2,1%	1,36 (0,96-1,95)
Women/Med-High inc /Born HIC/Age 18-34	1880	2,2%	1,48 (1,09-2,03)
Women/Low inc/Born Swe/Age 18-34	44237	2,9%	1,92 (1,77-2,09)
Men/Med-High inc/Born Swe/Age 35-49	61582	3,0%	2,00 (1,85-2,16)
Women/Low inc/Born HIC/Age 18-34	2243	3,1%	2,06 (1,61-2,64)
Men/Low inc/Born LMIC/Age 18-34	12578	3,3%	2,00 (1,85-2,16)
Women/Med-High inc/ Born LMIC/ Age 18-34	5181	4,0%	2,69 (2,31-3,14)
Men/ Med-High inc/ Born LMIC/Age 18-34	7534	4,1%	2,80 (2,46-3,19)
Women/Low inc/ Born LMIC/ Age 18-34	10538	4,3%	2,90 (2,59-3,25)
Men/Low inc/Born Swe/Age 35-49	21904	4,4%	2,99 (2,73-3,27)
Men/ Med-High inc/Born HIC/Age 35-49	3813	4,5%	3,03 (2,57-3,58)
Women/Med-High inc/Born Swe/Age 35-49	65512	4,7%	3,20 (2,97-3,45)
Women/Med-High inc/Born HIC/Age 35-49	4231	6,4%	4,46 (3,89-5,13)
Women/Low inc/Born Swe/Age 35-49	29863	7,0%	4,87 (4,50-5,26)
Men/ Med-High inc/Born Swe/Age 50-64	60552	7,4%	5,21 (4,85-5,60)
Men/Low inc/Born HIC/Age 35-49	2204	7,6%	5,33 (4,49-6,32)
Men/Med-High inc/Born LMIC/Age 35-49	8499	7,6%	5,34 (4,81-5,91)
Men/Low inc/Born LMIC/Age 35-49	13503	7,8%	5,49 (5,02-6,01)
Women/Low inc/ Born HIC/Age 35-49	2907	8,5%	6,06 (5,24-7,01)
Women/Med-High inc/ Born LMIC/Age 35-49	10097	9,5%	6,83 (6,23-7,50)
Men/Low inc/Born Swe/Age 50-64	7368	10,0%	7,21 (6,53-7,97)
Women/ Med-High inc /Born Swe/Age 50-64	64654	10,0%	7,22 (6,73-7,73)
Women/Low inc/Born LMIC/Age 35-49	14844	10,3%	7,44 (6,84-8,08)
Men/Med-High inc/Born HIC/Age 50-64	3582	10,7%	7,76 (6,85-8,78)
Men/Low inc/Born HIC/Age 50-64	960	12,8%	9,55 (7,82-11,66)
Women/ Med-High inc/Born HIC/Age 50-64	4517	13,3%	10,01 (8,99-11,14)
Women/Low inc/Born Swe/Age 50-64	10301	13,6%	10,24 (9,40-11,16)
Men/Low inc/Born LMIC/Age 50-64	4894	15,0%	11,50 (10,39-12,73)
Women/Low inc/Born LMIC/Age 50-64	4556	15,3%	11,75 (10,60-13,03)
Women/Low inc/Born HIC/Age 50-64	1253	15,4%	11,83 (10,02-13,97)
Men/Med-High inc/Born LMIC/Age 50-64	6197	16,5%	12,83 (11,69-14,08)
Women/Med-High inc/Born LMIC/Age 50-64	7466	17,0%	13,27 (12,15-14,49)

a Disposable income: Medium/High: 2nd and 3rd tertile, Low: 1st tertile.
b Country of birth: Sweden; HIC: High-income country; LMIC: Low-middle income country.
c Sick leave due to Covid-19.
d Odds ratios (ORs) and 95% confidence intervals (CI).
e Reference group.
