# Supplementary material for: The sociodemographic patterning of sick leave and determinants of longer sick leave after mild and severe COVID-19: a nationwide register-based study in Sweden
Source: Eur J Public Health. 2023 Oct 27;34(1):121–8. doi: 10.1093/eurpub/ckad191 (PMC10843940; doi:10.1093/eurpub/ckad191)
Supplement: ckad191_Supplementary_Data [file ckad191_supplementary_data.zip › ckad191_Supplementary_Data/ejph-2023-06-om-0328-File007.docx]

**Supplementary Figure 1** Daily numbers of individuals with a positive test for Sars-CoV-2 among gainfully employed individuals aged 18-64 years in the Swedish population from 1 January 2020 until September 2021 (yellow) and cumulative number of individuals on sick leave due to Covid-19 in the study population (red).
