# Supplementary figures and images for: The sociodemographic patterning of sick leave and determinants of longer sick leave after mild and severe COVID-19: a nationwide register-based study in Sweden
Source: Eur J Public Health. 2023 Oct 27;34(1):121–8. doi: 10.1093/eurpub/ckad191 (PMC10843940; doi:10.1093/eurpub/ckad191)

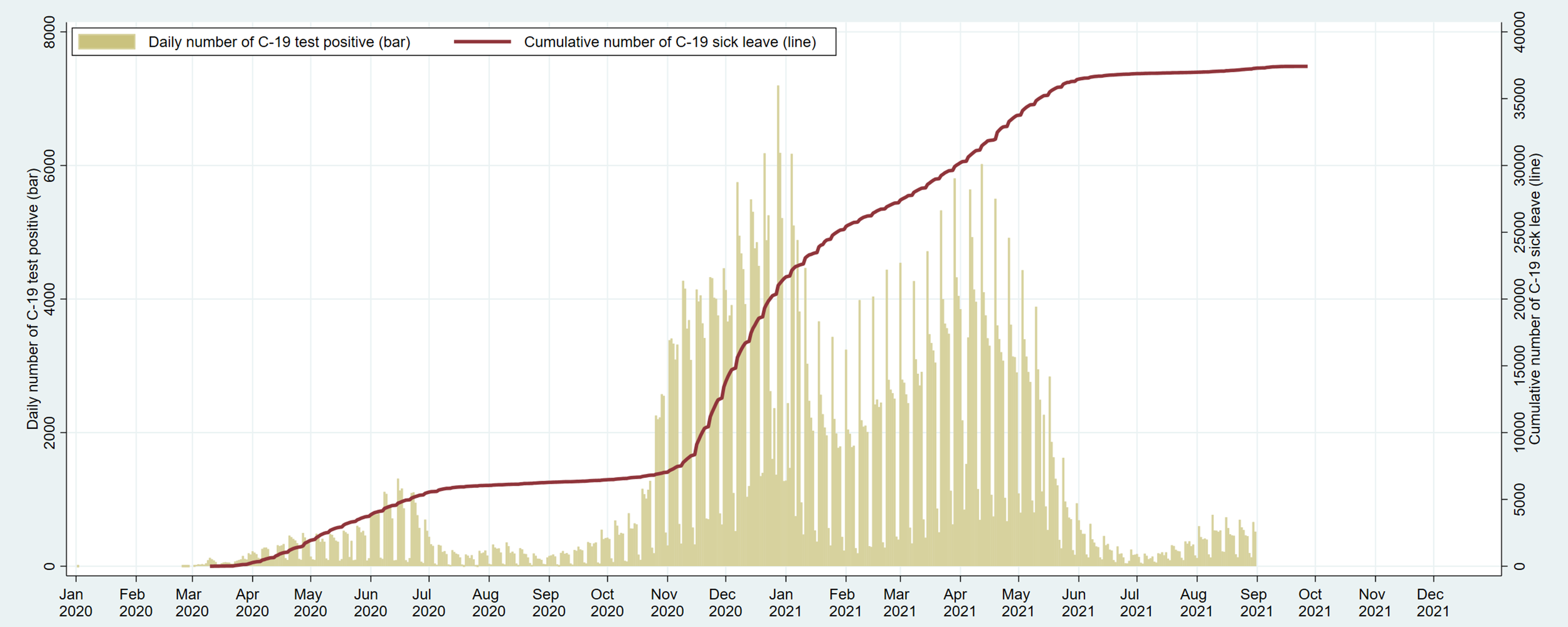

Supplement: ckad191_Supplementary_Data [file ckad191_supplementary_data.zip › ckad191_Supplementary_Data/ejph-2023-06-om-0328-File006.tif]
